# Functional Characterization of p.(Arg160Gln) PCSK9 Variant Accidentally Found in a Hypercholesterolemic Subject

**DOI:** 10.3390/ijms24043330

**Published:** 2023-02-07

**Authors:** Asier Larrea-Sebal, Chiara Trenti, Shifa Jebari-Benslaiman, Stefano Bertolini, Sebastiano Calandra, Emanuele A. Negri, Efrem Bonelli, Asier Benito-Vicente, Leire Uraga-Gracianteparaluceta, César Martín, Tommaso Fasano

**Affiliations:** 1 Department of Biochemistry and Molecular Biology, Universidad del País Vasco UPV/EHU, 48080 Bilbao, Spain; 2 Department of Molecular Biophysics, Biofisika Institute, University of Basque Country and Consejo Superior de Investigaciones Científicas (UPV/EHU, CSIC), 48940 Leioa, Spain; 3 Fundación Biofisika Bizkaia, 48940 Leioa, Spain; 4 Department of Internal Medicine Unit, Azienda USL—IRCCS di Reggio Emilia, 42122 Reggio Emilia, Italy; 5 Department of Internal Medicine, University of Genova, 16132 Genova, Italy; 6 Department of Biomedical, Metabolic and Neural Sciences, University of Modena and Reggio Emilia, 41125 Modena, Italy; 7 Clinical Pathology Unit, AUSL Romagna, 47521 Cesena, Italy

**Keywords:** familial hypercholesterolemia, PCSK9, GOF, LOF, characterisation, activity

## Abstract

Familial hypercholesterolaemia (FH) is an autosomal dominant dyslipidaemia, characterised by elevated LDL cholesterol (LDL-C) levels in the blood. Three main genes are involved in FH diagnosis: LDL receptor (LDLr), Apolipoprotein B (APOB) and Protein convertase subtilisin/kexin type 9 (PCSK9) with genetic mutations that led to reduced plasma LDL-C clearance. To date, several PCSK9 gain-of-function (GOF) variants causing FH have been described based on their increased ability to degrade LDLr. On the other hand, mutations that reduce the activity of PCSK9 on LDLr degradation have been described as loss-of-function (LOF) variants. It is therefore important to functionally characterise PCSK9 variants in order to support the genetic diagnosis of FH. The aim of this work is to functionally characterise the p.(Arg160Gln) PCSK9 variant found in a subject suspected to have FH. Different techniques have been combined to determine efficiency of the autocatalytic cleavage, protein expression, effect of the variant on LDLr activity and affinity of the PCSK9 variant for the LDLr. Expression and processing of the p.(Arg160Gln) variant had a result similar to that of WT PCSK9. The effect of p.(Arg160Gln) PCSK9 on LDLr activity is lower than WT PCSK9, with higher values of LDL internalisation (13%) and p.(Arg160Gln) PCSK9 affinity for the LDLr is lower than WT, EC50 8.6 ± 0.8 and 25.9 ± 0.7, respectively. The p.(Arg160Gln) PCSK9 variant is a LOF PCSK9 whose loss of activity is caused by a displacement of the PCSK9 P’ helix, which reduces the stability of the LDLr-PCSK9 complex.

## 1. Introduction

Familial hypercholesterolaemia (FH) is an autosomal dominant dyslipidaemia, characterised by elevated LDL cholesterol (LDL-C) levels in the blood. Heterozygous familial hypercholesterolaemia is one of the more common genetic diseases with a prevalence of 1:250–300 in the general population [1]. FH patients have a high probability of developing cardiovascular disease, due to atherosclerotic lesions in the arterial endothelium caused by high plasma LDL-C concentrations. Regarding the aetiology of the disease, mutations in three genes involved in cholesterol metabolism are mainly responsible for the development of the disease: *LDL receptor* (*LDLr*), *Apolipoprotein B* (*APOB*) and *Protein convertase subtilisin/kexin type 9* (*PCSK9*) [2]. Genetic alterations that occur in these genes reduce plasma LDL-C clearance by different mechanisms. Mutations in the *LDLR,* responsible for LDL-C clearance from plasma, have been detected in the majority of FH patients (85%). On the other hand, mutations in *APOB*, which are responsible for 5–10% of FH cases, impair LDL–LDLr binding, thus leading to LDL-C accumulation in the plasma [2]. Finally, gain-of-function (GOF) mutations in PCSK9, a protein involved in the fine-tuning of LDLr expression at the plasma membrane, account for 1% of FH cases by an increased lysosomal degradation of the LDLr [3].

The human *PCSK9* gene is located in the 1p32.3 region of the short arm of the first chromosome. The *PCSK9* gene, a 22 kb sequence consisting of 12 exons and 11 introns, encodes a protein of 692 amino acids [4]. PCSK9 expression is transcriptionally regulated by the sterol-regulatory element-binding protein (SREBP2), which regulates cholesterol metabolism. In fact, when the intracellular concentration of LDL-C is low, SREBP2 induces *LDLR* transcription together with *PCSK9* [5]. The protein is distributed in five domains: N-terminal signal peptide (SP; 1–30 aa), propeptide or inhibitory prodomain (31–152 aa), subtilisin/serin protease-like catalytic domain (SCD; 153–451 aa) and the C-terminal cysteine- and histidine-rich domain (CHRD; 452–692 aa) [6]. PCSK9 is synthesised as a 74 kDa precursor, which becomes mature after an autocatalytic cleavage between residues 152–153 rendering a 14 kDa prodomain, which binds non-covalently to the 60 kDa catalytic domain [6]. The autocatalytic cleavage is essential for protein secretion and also results in catalytic activity inhibition by hiding the catalytic triad (D186, H226 and S386) [7].

Once secreted, PCSK9 binds to the EGF-A domain of the LDLr, and the complex is endocytosed via clathrin-coated vesicles. Acidification of the endosome increases the affinity of the protein for the receptor that targets the PCSK9-LDLr complex to the lysosome leading to LDLr degradation [8].

Basal PCSK9 activity is necessary to maintain a balance in membrane LDLr levels, thus allowing an adequate degradation/recycling ratio. To date, several PCSK9 GOF variants that cause autosomal dominant hypercholesterolaemia (ADH) by reducing LDLr levels have been described [9]. On the other hand, LOF mutations that reduce the activity of PCSK9, showing an impaired ability to degrade LDLr have also been described [9,10,11]. It is therefore important to functionally characterise PCSK9 variants to validate the genetic diagnosis of ADH.

The aim of this work is to functionally characterise the activity of the p.(Arg160Gln) PCSK9 variant, a variant accidentally found in a hypercholesterolemic patient. In order to carry out the functional characterisation, biophysics and molecular biology techniques have been combined to determine the efficiency of the autocatalytic cleavage, protein expression, the effect of p.(Arg160Gln) PCSK9 on LDLr activity and the affinity of p.(Arg160Gln) PCSK9 variant for the LDLr. To understand the mechanism by which p.(Arg160Gln) presents a reduced affinity for the LDLr, a computational study using AlphaFold has been used.

## 2. Results

**Figure 1:**
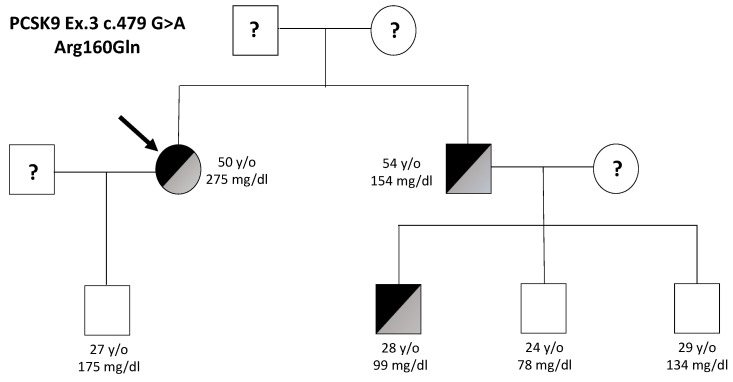
Family pedigree showing the carriers of the p.(Arg160Gln) PCSK9 variant. Half-blackened indicates heterozygous carriers of the p.(Arg160Gln) variant. The arrow represents the proband. Age (in years) and LDL-C (mg/dL) are given. “?” unknown.

Next-generation sequencing (NGS) analysis was employed to sequence genes associated with Familial Hypercholesterolemia in the proband’s sample. A missense PCSK9 mutation was identified: c.479 G > A in exon 3, p.(Arg160Gln). Subsequent cascade screening led to the identification of the same PCSK9 mutation in two of the five family members that were subjected to genetic analysis. (Figure 1). Lipid values of family members and PCSK9 genotypes are reported in Table 1. The information about lipid profiles obtained with the family pedigree demonstrated no clear correlation between the PCSK9 mutation and the high LDL-C phenotype as one of the carriers of the mutation had normal–low LDL-C values. A high LDL-C phenotype with the p.(Arg160Gln) variant was present only in one of the three mutation carriers., which is not expected for a GOF PCSK9 variant. The ClinVar database describes the frequency of the identified PCSK9 mutation (c.479 G > A, p.Arg160Gln) of 0.00013 as it was found in 19/282782 chromosomes in the general population (data from the Genome Aggregation Database, gnomAD). The mutation was reported in three subjects with suspected autosomal dominant hypercholesterolemia and in one subject with hypobetalipoproteinemia (ClinVar database). The variant has been also reported in the literature in a GWAS study observed in one individual with low LDL-C [12]. The available evidence was not sufficient to determine the role of this variant in disease conclusively. For that reason, we decided to functionally characterise the effect on the PCSK9 protein.

We analysed the potential consequences of the p.(Arg160Gln) PCSK9 variant with the in silico prediction programs Mutation Taster, Polyphen-2 PROVEAN; SIFT, Mutation Assessor, FATHMM and PANTHER. The variant was classified as a probably damaging to disease causing PCSK9 variant (Table 2). To our knowledge, no experimental evidence studying the impact of the mutation on protein function has been reported.

**Table 1:** Sex, age, lipid profiles and mutational status of proband and family members characterised during the study. TC, LDL-C, HDL-C and TG are expressed in mg/dL.

Subject	Sex	Age	TC	LDL-C	HDL-C	TG	p.(Arg160Gln)
Proband	F	50	341	275	59	139	YES
Son	M	27	282	175	76	199	NO
Brother	M	54	200	154	39	98	YES
Nephew 1	M	28	169	99	53	88	YES
Nephew 2	M	24	136	78	51	51	NO
Nephew 3	M	29	187	134	44	132	NO

**Table 2:** In silico predictions of the impact of the Arg160Gln mutation on PCSK9 protein using different online tools.

	Mutation Taster	PolyPhen-2	PROVEAN	SIFT	Mutation Assessor	FATHMM	PANTHER
p.(Arg160Gln)	Disease Causing	Probably damaging	Deleterious	Damaging	High Impact	Damaging	Probably Damaging

### 2.1. PCSK9 Expression, Maturation and Secretion

**Figure 2:**
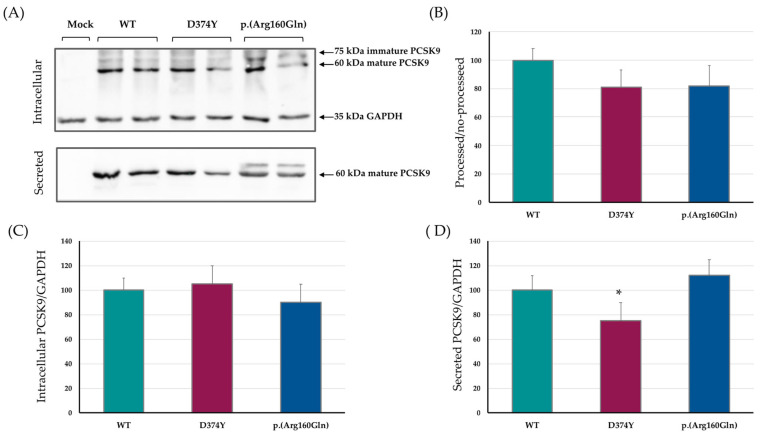
Expression, maturation and secretion of the p.(Arg160Gln) PCSK9 variant is similar to WT PCSK9. (**A**) Representative immunoblots of expression and secretion of PCSK9 on HEK293 cells transiently transfected with mock (empty plasmid), WT, D374Y and p.(Arg160Gln). (**B**) Ratio between processed/non-processed PCSK9 quantified by densitometry. (**C**) Amount of expressed PCSK9 determined as the ratio between intracellular PCSK9/GAPDH quantified by densitometry. (**D**) Amount of secreted PCSK9 determined as the ratio between media/GAPDH quantified by densitometry. Histograms represent the mean ± SD of three independent experiments. * *p* < 0.05 compared to WT PCSK9.

Expression, maturation and secretion of the WT, D374Y and p.(Arg160Gln) PCSK9 variants were studied in transiently transfected HEK293 cells, a well-established cell line, to perform PCSK9 functional studies [9,13,14,15]. As shown in Figure 2, the p.(Arg160Gln) PCSK9 variant showed similar expression, maturation and secretion patterns than WT PCSK9 as detected by Western blot. The p.(Arg160Gln) variant showed a faint extra-band of slightly higher molecular weight in the culture media. In terms of total PCSK9 expression and secretion ratios, no significant changes were observed (Figure 2B–D).

### 2.2. Effect of the p.(Arg160Gln) PCSK9 Variant on LDL Uptake

**Figure 3:**
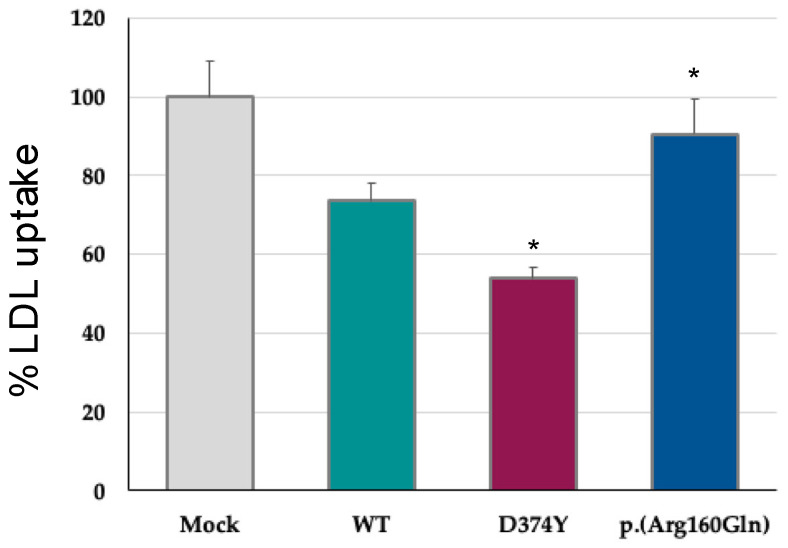
Expression of the p.(Arg160Gln) PCSK9 variant increases LDL uptake compared to WT. Transiently transfected HEK293 cells with the different PCSK9 variants were incubated with FITC-labelled LDL and lipoprotein uptake was measured by flow cytometry. Histograms represent the mean ± SD of three independent experiments. * *p* < 0.01 compared to WT. Mock corresponds to an empty plasmid. LDL uptake was normalised to the total amount of PKSC9 secreted to the culture medium 48 h post-transfection.

Analysis of LDLr activity was assessed in transiently transfected HEK293 cells with the PCSK9 variant, and LDL uptake was determined by flow cytometry as described in Methods. As shown in Figure 3, cells expressing the p.(Arg160Gln) variant show a significant higher LDL uptake compared to WT PCSK9.

### 2.3. p.(Arg160Gln) PCSK9 Affinity (EC50) for the LDLr

**Figure 4:**
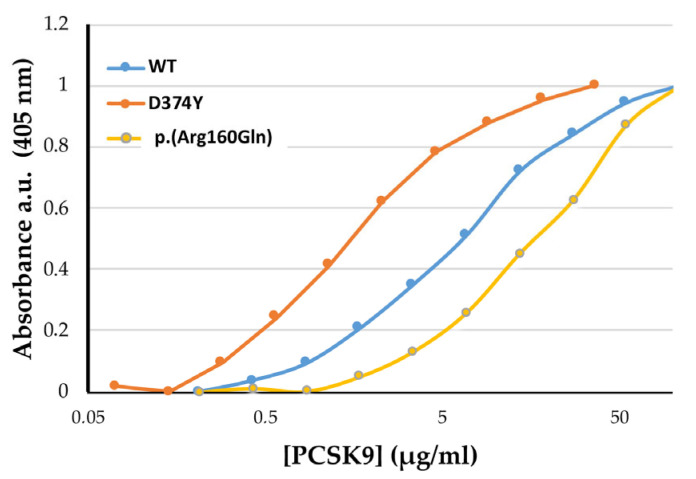
The p.(Arg160Gln) PCSK9 variant shows lower affinity to the LDLr compared to WT. Affinity curves representing the binding affinity of PCSK9 variants for the LDLr determined by solid-phase immunoassay at pH 7.4. Data represents the means of three independent experiments. EC_50_ values are shown in Table 3.

We next assessed the affinity of the p.(Arg160Gln) PCSK9 variant for the LDLr. Binding affinities were determined by solid-phase immunoassay as described in Methods. As shown in Table 3 and Figure 4, the affinity of p.(Arg160Gln) PCSK9 for the LDLr was significantly reduced compared to that of WT.

**Table 3:** EC_50_ of the WT, D374Y GOF PCSK9 variant and the p.(Arg160Gln) PCSK9 variant.

	WT	D374Y	p.(Arg160Gln)
EC_50_	8.57 ± 0.76	1.54 ± 0.38 *	25.96 ± 0.72 *

* *p* < 0.01 compared to WT.

### 2.4. Bioinformatic Analysis of p.(Arg160Gln) PCSK9 3D Structure

**Figure 5:**
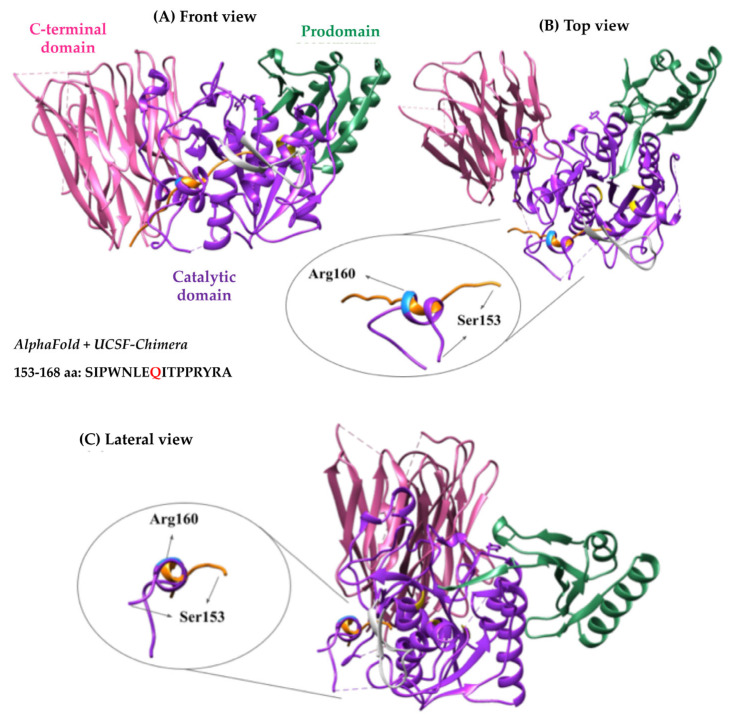
AlphaFold-2 structure prediction of the p.(Arg160Gln) PCSK9 variant compared to WT. Structures of the area of interest (amino acids 153–160) in both WT and p.(Arg160Gln) were compared to understand the nature of the variant. (**A**) Front view, (**B**) top view and (**C**) side view. In green, prodomain (31–152); in purple, catalytic domain (154–451); in pink, C-terminal domain (453–692). Inside the catalytic domain, Arg160 residue is coloured in blue, the catalytic triad in yellow (186, 226 and 386) and the LDLr binding site in grey (367–381).

Using AlphaFold-2, the 3D structures of WT and p.(Arg160Gln) PCSK9 were compared. Upon structure alignment, the amino acids preceding the mutation site (153–160) were displaced in the variant (Figure 5). According to the modelling, the replacement of an Arg by a Gln causes a displacement of the amino acids at positions 153–156 so that they move away from the catalytic domain of PCSK9, a key factor in the interaction with the LDLr’s EGF-A domain (Figure 5).

## 3. Discussion

Heterozygous Familial Hypercholesterolemia is the most common genetic disease, which requires early detection and appropriate treatment to reduce cardiovascular events [1]. In recent years, and thanks to massive sequencing of DNA, many variants of genes involved in the development of FH have been identified; however, not all of them are pathogenic [1,9,16,17]. Although genetic analyses constitute an important step for diagnosis, establishing the pathogenicity of these variants through functional studies is essential to make an accurate diagnosis [18].

In the present work we have functionally characterised the effect of the p.(Arg160Gln) PCSK9 variant to find out whether its function is altered or not. This variant was identified in a 50 y/o female in postmenopausal state with high LDL-C levels. Cascade screening in five family members was not conclusive to establish the supposed “gain-of-function” of the mutated PCSK9 protein. In particular the mutation was identified in a 28 y/o male with normal/low LDL-C. In silico prediction of the effect of the mutation supported a deleterious impact of the mutation on the protein function, but, for obvious reason, without giving advice on gain or loss of protein function. For the p.(Arg160Gln) mutation, a very low frequency was described in genomic databases and it was identified in both hyper- and hypo-cholesterolemic conditions. The available evidence was considered insufficient to determine the role of this variant in disease conclusively. Therefore, this variant was classified as a variant of uncertain significance, claiming that its association with disease require further investigation.

The p.(Arg160Gln) mutation is located at the beginning of the PCSK9-catalytic domain, as shown in the present work; expression/processing and secretion of the variant is not affected. Interestingly, the higher values of LDL internalisation in cells expressing p.(Arg160Gln) indicates that the activity of p.(Arg160Gln) on LDLr activity is lower compared to WT PCSK9. Therefore, the functional characterisation performed in this work indicates that p.(Arg160Gln) PCSK9 is a LOF variant, thus, is not responsible of the patient’s phenotype. In addition, the LOF activity of the p.(Arg160Gln) variant was corroborated by determining its affinity for the LDLr, which showed a reduced EC_50_ for the LDLr compared to that of WT PCSK9.

**Figure 6:**
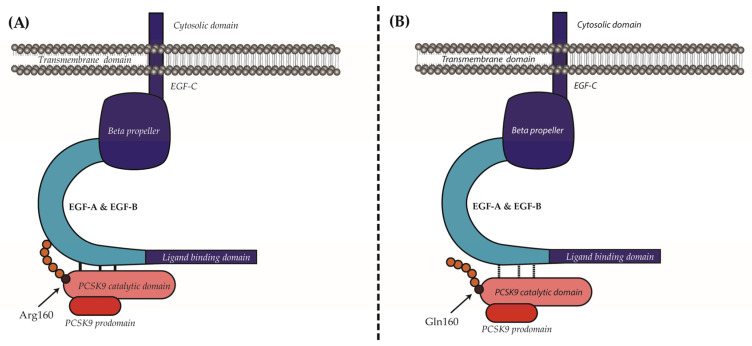
Suggested mechanisms leading to loss of affinity of the p.(Ard160Gln) PCSK9 variant for the LDLr. (**A**) Upon prodomain cleavage, P′ helix is located next to the LDLr-binding site and strengthens the electrostatic forces for binding and maintaining the PCSK9-LDLr complex. Additionally, the Arg160 residue forms a salt bridge with the LDLr Asp343 residue. (**B**) Replacement of Arg160 by a Gln modifies the change the direction of rotation of the P’ helix, which no longer remains close to the EGF-A residues implicated in PCSK9 binding.

We next sought to understand the mechanism by which the p.(Arg160Gln) variant shows a reduced affinity for the LDLr. It has been described that autocatalysis is critical for PCSK9 secretion and endows LDLr-binding capacity by exposing the EGF(A)-binding site [19]. The autocatalytic cleavage occurs between the last prodomain residue, Gln152 (P1 residue; nomenclature of Schechter and Berger) and the first residue of the catalytic domain Ser153 (P1′ residue). Once cleaved, a spring-loaded mechanism is triggered and P′ amino acids translocate into a groove located next to the LDLr-binding site (Figure 6). The EGF(A)-interacting site of PCSK9 is a solvent-exposed area of ~530 Å2 that is largely flat, featureless and devoid of binding pockets. Therefore, if electrostatic forces are not strong enough, binding and maintaining the PCSK9-LDLr complex could be compromised. Interestingly, the stretch of P′ residues (Ser153–Thr162) adopts an α-helical conformation, termed the P′ helix, in which P1′ Ser153 and P3′ Pro155 contribute to the stabilisation of the bound LDLr-EGF(A) domain through polar and van der Waals interactions [20,21]. In addition, the Arg160 residue contributes specifically to binding affinity and specificity by forming a salt bridge with the LDLr Asp343 residue [19]. As Gln has no charge, replacement of the positively charged Arg160 by Gln abrogates the interaction with the negatively charged Asp343 of the LDLr. Hence, the loss of this electrostatic interaction can contribute to the reduced affinity determined here of the p.(Arg160Gln) PCSK9 variant for the LDLr. By using AlphaFold, we have also determined that replacement of Arg160 by Gln affects the conformation of the 153–168 P’ residues. P’ helix modelling shows that the substitution causes a change in helix rotation, which results in a complete displacement of the amino acids involved in the stabilisation of PCSK9-EGF-A binding (Figure 5 and Figure 6). It seems plausible that this predicted displacement of the PCSK9 P’ helix in p.(Arg160Gln) variant represents the main reason for the lower affinity of the variant for the LDLr.

According to the obtained results, we can conclude that the p.(Arg160Gln) PCSK9 variant is a LOF PCSK9 variant whose loss of activity is caused by a displacement of the PCSK9 P’ helix, which reduces the stability of the LDLr-PCSK9 complex, thus not causative of familial hypercholesterolemia.

## 4. Materials and Methods

### 4.1. Patients

A 50 y/o postmenopausal female was presented to the Lipid Clinic for the finding of very high LDL-C cholesterol (Table 1) and a family history of hypercholesterolemia and treatment with lipid-lowering agents. The diagnosis of probable Familial Hypercholesterolemia was made based on a Dutch Lipid Clinical Network (DLCN) (Ref) score of 6. Next-generation sequencing (NGS) analysis was performed using the Illumina MiSeq DX platform (Illumina, San Diego, CA, USA) to sequence genes associated with familial hypercholesterolemia (APOB, APOE, LDLR, LDLRAP1, PCSK9, ABCG5, ABCG8, CYP27A1, LIPA, MYLIP) [22]. Genetic analysis was also performed on five family members (son, brother and three nephews). Lipid profiles of proband and family members are reported in Table 1.

### 4.2. Sample Analysis

Total cholesterol (TC), HDL-C, LDL-C and triglycerides (TG) were measured using fully automatic laboratory instrumentation with enzymatic colorimetric assays (Atellica CH, Siemens Healthineers, Erlangen, Germany).

For all subjects involved in the study, samples for lipid profiles were obtained after an overnight fasting period and without administration of lipid-lowering drugs. As shown in Figure 1 and Table 1, two mutation carriers had high or mild elevation of plasmatic LDL-C values (275 mg/dL and 154 mg/dL in the proband and the brother, respectively). Third mutation carrier (Nephew 1) had normal-low LDL-C values (99 mg/dL).

### 4.3. Site-Directed Mutagenesis and Cloning

Plasmids carrying PCSK9 variants were constructed by Innoprot. Mutations were introduced into the human PCSK9 cDNA (NM_174936.3), in the mammalian expression vector WT-PCSK9 plasmid (pCMV-PCSK9-FLAG) by oligonucleotide site-directed mutagenesis, using the QuickChange Lightning mutagenesis kit (Agilent Technologies Inc., La Jolla, CA, USA) according to the manufacturer’s instructions. This vector contains a 6× His tag for the purification and a FLAG epitope (DYKDDDDK) as a specific target for antibodies. Restriction enzyme digestion of the appropriate fragments and the integrity of the remaining PCSK9 cDNA sequences of all constructs were verified by direct sequence analysis.

### 4.4. PCSK9 Expression on HEK293 Cells

A total of 5 × 105 HEK293 cells were transfected with an empty plasmid or with 1.5 µg of a plasmid encoding WT-PCSK9, GOF-PCSK9 p.(Asp374Tyr) or the analysed variant p.(Arg160Gln) using a Calcium Phosphate Transfection Kit (Invitrogen, Thermo Fisher Scientific, Pierce, CA, USA). The next day cells were washed and incubated with DMEM medium containing 10% FBS, 2 mM L-Glutamine and antibiotics (100 units/mL penicillin; 100 μg/mL streptomycin) (complete medium) for 48 h. Next, cell supernatants were collected and cells were lysated to analyse both PCSK9 secretion and expression.

### 4.5. PCSK9 Secretion and Expression Analysis by Western Blot

PCSK9 expression and secretion analysis in HEK293 cells transfected with empty plasmid and the different PCSK9 variants was performed by Western blotting. For that purpose, proteins from cell lysates or the supernatants were resolved by 8.5% Tris-Glycine SDS-PAGE. Gels were next blotted onto Nitrocellulose membranes (Protran BA 83, Whatman™, GE Healthcare, Munich, Germany), blocked for 1 h in TBS (50 mM Tris-HCl, pH 7.5, 150 mM NaCl, 0.1% Tween 20) containing 5% BSA and immunoblotted with a rat monoclonal anti-FLAG antibody (1:1000) (Cat. No:MA1–142; Invitrogen, Thermo Fisher Scientific, Pierce, CA, USA) for 16 h at 4 °C. Then, they were counterstained with a horseradish peroxidase-conjugated goat anti-rat antibody (Cat. No: 7077S; Cell Signalling Technology^®^ Inc., Danvers, MA, USA). The signal was created by adding SuperSignal West Dura Extended Substrate (Thermo Fisher Scientific, Pierce, CA, USA) and it was measured by the ChemiDoc XRS chemiluminescence system (Bio-Rad, Hercules, CA, USA) at 425 nm. The relativisation was performed using Glyceraldehide 3-phosphate dehydrogenase (GAPDH) as control.

### 4.6. Activity of PCKS9 Variants by Flow Cytometry

#### 4.6.1. LDL Labelling with Fluorescein Isothiocyanate (FITC)

LDL was purified from blood plasma by centrifugation at 120,000× *g* at 4 °C for 19 h. LDL (1.019–1.050 g/mL) was isolated through isopycnic ultracentrifugation by adjusting plasma density to 1.21 g/mL by the addition of KBr. LDL particles were labelled with FITC as previously described [23]. Briefly, 10 μL of FITC (2 mg/mL) were added to 1 mL LDL (1 mg/mL apoB) in 0.1 M NaHCO3, pH 9.0, and mixed for 2 h by slow rocking at room temperature. The unreacted dye was removed by gel filtration on a Sephadex G-25 column equilibrated with PBS EDTA-free buffer. All fractions were assayed for protein content using bovine serum albumin as standard (Pierce BCA protein assay; Pierce, Thermo Fisher Scientific, Pierce, CA, USA).

#### 4.6.2. Flow Cytometry

HEK293 cells were transfected with the vector containing PCSK9 variants as explained previously, and they were incubated with 20 μg/mL of FITC-labelled LDL for 4 h prior to the experiment. After the incubation, the internalised LDL was measured by fluorescence-activated cell sorter (FACS) as described before [23]. Fluorescence was measured by FACSCalibur™ (BD Bioscience, San Jose, CA, USA). Each sample was triplicated and 10,000 events were measured in each case.

#### 4.6.3. PCSK9 Quantification

Aliquots from the culture mediums were collected at 48 h and PCSK9 levels were determined by ELISA following manufacturer instructions (Quantikine^®^ ELISA; R&D Systems, McKinley Place, MI, USA, Cat. No: DPC900)

#### 4.6.4. Solid-Phase Immunoassay for PCSK9-LDLr Ectodomain Affinity

- PCSK9 purification from stably transfected HEK293

HEK293 cells grown to sub-confluence were transfected with the different PCSK9 plasmids and selected with geneticin (G418 sulphate) (Gibco, Thermo Fisher Scientific, Pierce, CA, USA) according to the manufacturer’s instructions to obtain stably transfected cells. For PCSK9 purification, stably transfected HEK293 cells were grown at 80% confluence in complete DMEM medium. Then, the culture medium was replaced by Opti-MEM (Invitrogen, Thermo Fisher Scientific, Pierce, CA, USA) without geneticin and cells were maintained under these conditions for 48 h. Finally, the medium was harvested and PCSK9 was purified using one-step nickel affinity chromatography. Purified PCSK9 variants were stored at −80 °C in 50 mM Tris-HCl buffer supplemented with 150 mM NaCl and 10% glycerol, pH 8.0.

- LDLr-ectodomain production and purification

The LDLr construct encoding the N-terminal extracellular ectodomain (ED-LDLr, corresponding to 1–789 amino acids) and His tags was purified by affinity chromatography from cells transfected with the pcDNA3.1-EC-LDLR-His plasmid, kindly provided by Prof. Leren. Briefly, HEK293 cells at 70–80% confluence were transfected with the plasmid by calcium phosphate method for 24–48 h and selected in successive passages by geneticin (G-418 sulphate; Gibco, Thermo Fisher Scientific, Pierce, CA, USA). For ED-LDLr expression and purification, the culture medium of transfected cells was changed to Opti-MEM (Invitrogen, Thermo Fisher Scientific, Pierce, CA, USA) without geneticin and maintained under these conditions for three additional days. Then the medium was harvested, supplemented with protease inhibitors (cOmplete™ EDTA-free; Roche, Merck, Germany) and the ED-LDLr was affinity purified using one-step nickel affinity chromatography. For protein long-term maintenance, the buffer was changed to storage buffer (50 mM Tris-HCl, 50 mM NaCl, 10% glycerol, and 0.01% Brij-35, pH 7.5) and frozen at −80 °C.

- ELISA assay

LDLr ectodomain fragments diluted in working buffer (10 mM TrisHCl, 50 mM NaCl, 2 mM CaCl_2_, pH 7.4) were coated at 0.4 μg/mL onto 96-well microtiter plates by incubation overnight at 4 °C. Plates were then blocked with working buffer supplemented with 5% (*w*/*v*) BSA and incubated with a serial dilution of each of the different PCSK9 variants diluted in working buffer at pH 7.4 for 2 h at room temperature, and then washed thoroughly with working buffer supplemented with 0.1% (*w*/*v*) Tween 20 (SigmaAldrich, Merck, Germany). For ligand detection, the antibodies, rat monoclonal anti-DYKDDDDK tag and peroxidase-conjugated goat anti-rat were diluted in working buffer supplemented with 5% (*w*/*v*) BSA, applied directly to the plate and incubated for 1 h at room temperature, with an extensive washing between both incubations. After a final wash, antibody binding was determined using 50 μL per well of 2,2′-Azino-bis (3-ethylbenzothiazoline-6-sulfonic acid) substrate solution (Sigma-Aldrich, Merck, Germany) and measuring colour change at 405 nm. The time course for colour development was essentially linear and measurements were taken 30–60 min after the addition of substrate. For data processing, all absorbance values were corrected for unspecific binding, relativised to maximum absorbance and EC50 values were extracted from curves after fitting the data to a 5-parameter logistic (5-PL) equation (SigmaPlot 13.0, Systat Software Inc., San Jose, CA, USA).

### 4.7. PCSK9 3D Structure Bioinformatics Analysis

PCSK9 structure was obtained from PBD (PDB ID: 2p4e) and the effect of the mutation on the structure was analysed using AlphaFold ColabFold 2. Both the complete protein and a short sequence that comprised the mutation site were analysed with the software. The obtained alternative structure was compared to WT.

### 4.8. Statistical Analysis

Data are presented as mean ± SD or means (interquartile range) for continuous variables. Experimental data presented in graphs are reported as percentage relative to the control. Flow cytometry and solid-phase immunoassays were performed independently 3 times and all measurements were performed in triplicate. Western blots from 3 independent experiments were quantified by densitometry to determine statistical significance.

The Shapiro–Wilk test was performed to confirm that the data were normally distributed and F test for equality of variances. The null hypothesis was verified, indicating that the data were normally distributed. A 2-tailed Student t test with a significance level of 0.05 was used to test for differences in PCSK9 activity between WT and p.(Arg160Gln) PCSK9 variant. All statistical analyses were performed with the Prism software (GraphPad Prism version 8 for Mac, GraphPad Software, La Jolla, CA, USA, www.graphpad.com, accessed on 1 September 2022.

### 4.9. Study Limitation

In this study, Student’s *t*-test has been performed with N = 3. For sample size N ≤ 5 per group, it is recommended either to perform a nonparametric statistical test or to increase sample size.

## Data Availability

All data are available upon request to the corresponding authors.
